# Smelly interactions: host-borne volatile organic compounds triggering behavioural responses in mosquitoes, sand flies, and ticks

**DOI:** 10.1186/s13071-024-06299-1

**Published:** 2024-05-16

**Authors:** Marcos Antonio Bezerra-Santos, Giovanni Benelli, Giacinto Salvatore Germinara, Petr Volf, Domenico Otranto

**Affiliations:** 1 Department of Veterinary Medicine, University of Bari, Bari, Italy; 2 Department of Agriculture, Food and Environment, University of Pisa, Pisa, Italy; 3 Dipartimento di Scienze Agrarie, Alimenti, Risorse Naturali e Ingegneria, University of Foggia, Foggia, Italy; 4 Department of Parasitology, Faculty of Science, Charles University, Prague, Czech Republic; 5 Department of Veterinary Clinical Sciences, City University of Hong Kong, Hong Kong, China

**Keywords:** Attraction, Host searching, Insect vectors, Questing, Repellent, Vector-borne pathogens, VOCs

## Abstract

Volatile organic compounds (VOCs) are chemicals emitted as products of cell metabolism, which reflects the physiological and pathological conditions of any living organisms. These compounds play a key role as olfactory cues for arthropod vectors such as mosquitoes, sand flies, and ticks, which act in the transmission of pathogens to many animal species, including humans. Some VOCs may influence arthropod behaviour, e.g., host preference and oviposition site selection for gravid females. Furthermore, deadly vector-borne pathogens such as *Plasmodium falciparum* and *Leishmania infantum* are suggested to manipulate the VOCs profile of the host to make them more attractive to mosquitoes and sand fly vectors, respectively. Under the above circumstances, studies on these compounds have demonstrated their potential usefulness for investigating the behavioural response of mosquitoes, sand flies, and ticks toward their vertebrate hosts, as well as potential tools for diagnosis of vector-borne diseases (VBDs). Herein, we provide an account for scientific data available on VOCs to study the host seeking behaviour of arthropod vectors, and their usefulness as attractants, repellents, or tools for an early diagnosis of VBDs.

**Graphical Abstract:**

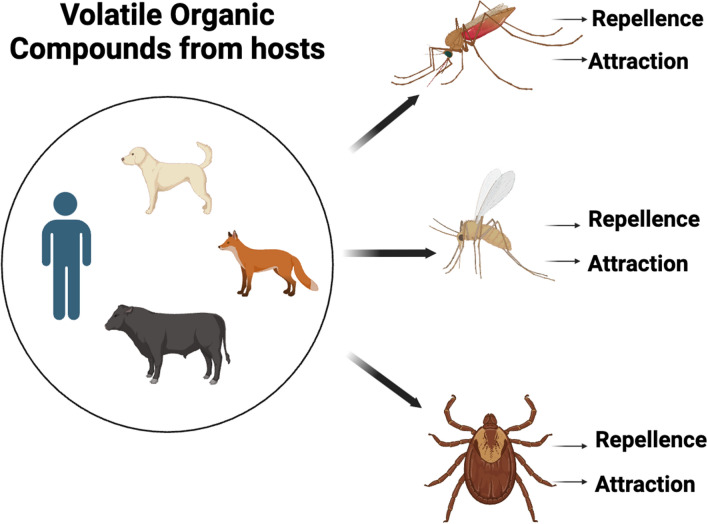

## Background

Mosquitoes, ticks, and sand flies are among the most important vectors of pathogens worldwide, being responsible for the transmission of infectious agents such as viruses, bacteria, nematodes, and protozoa of human and veterinary concern [1–3]. The mechanisms used by these arthropod vectors to locate susceptible vertebrate hosts include visual, olfactory, acoustic, and thermal stimuli. Such mechanisms influence the host seeking behaviour of individual arthropods according to the species characteristics and biology, even within the same taxonomic group. For example, many species of ticks (e.g., *Dermacentor* spp., *Ixodes* spp., *Rhipicephalus* spp.) have a questing behaviour, in which they climb the vegetation and wait for the appropriate host, whereas other species such as *Hyalomma* spp. are considered hunting ticks, since they actively chase for vertebrate hosts [4–6]. Another example is the South American sand fly species *Lutzomyia* (*Lutzomyia*) *longipalpis,* in which females are attracted not only by the host odour, but also by aggregation of sex pheromones of sand fly males that arrive first to the host [7–10].

Among the cues used by arthropods, the olfactory stimuli have been the most studied, being crucial to identify the preferential hosts, principally for vectors with a high host specificity [11, 12]. Volatile organic compounds (VOCs) are a group of chemicals in a gaseous phase and with high vapor pressure, which are delivered as products of the cell metabolism, reflecting the physiological and pathological conditions of the organisms [13]. The US Environmental Protection Agency (EPA; https://www.epa.gov/) define VOCs as compounds of carbon, excluding gases that participate in atmospheric photochemical reactions, such as carbon monoxide, carbon dioxide (considered an universal attractant for arthropods), carbonic acid, metallic carbides or carbonates, and ammonium carbonate. Importantly, some VOCs affect the behaviour of arthropod vectors, which indicates that studying these molecules is crucial to understand the host preference of mosquitoes, sand flies, and ticks [14–17]. In addition, such compounds may be applied to improve the trapping methods available for capturing these arthropods [11, 18, 19].

Several VOCs are emitted by vertebrate hosts. However, a relatively small amount of them have an influence on the arthropod behaviour, being defined as ‘allelochemicals’ [11]. The latter include kairomones (attractants) and allomones (repellents) [11]. Kairomones may be applied as selective tools for studying population abundance, surveillance of invasive species and vector-borne pathogens, as well as for predicting pathogen outbreaks [20, 21]. Conversely, the repellent effect of allomones emitted by fungi, bacteria, yeasts, plants, and mammals represents a potential tool for controlling arthropod vectors [22–25]. The presence of VOCs has also been explored in the diagnosis of cancer and infectious diseases in humans and animals [13, 26–28], as well as for managing agricultural pests [21, 29, 30].

**Fig. 1:**
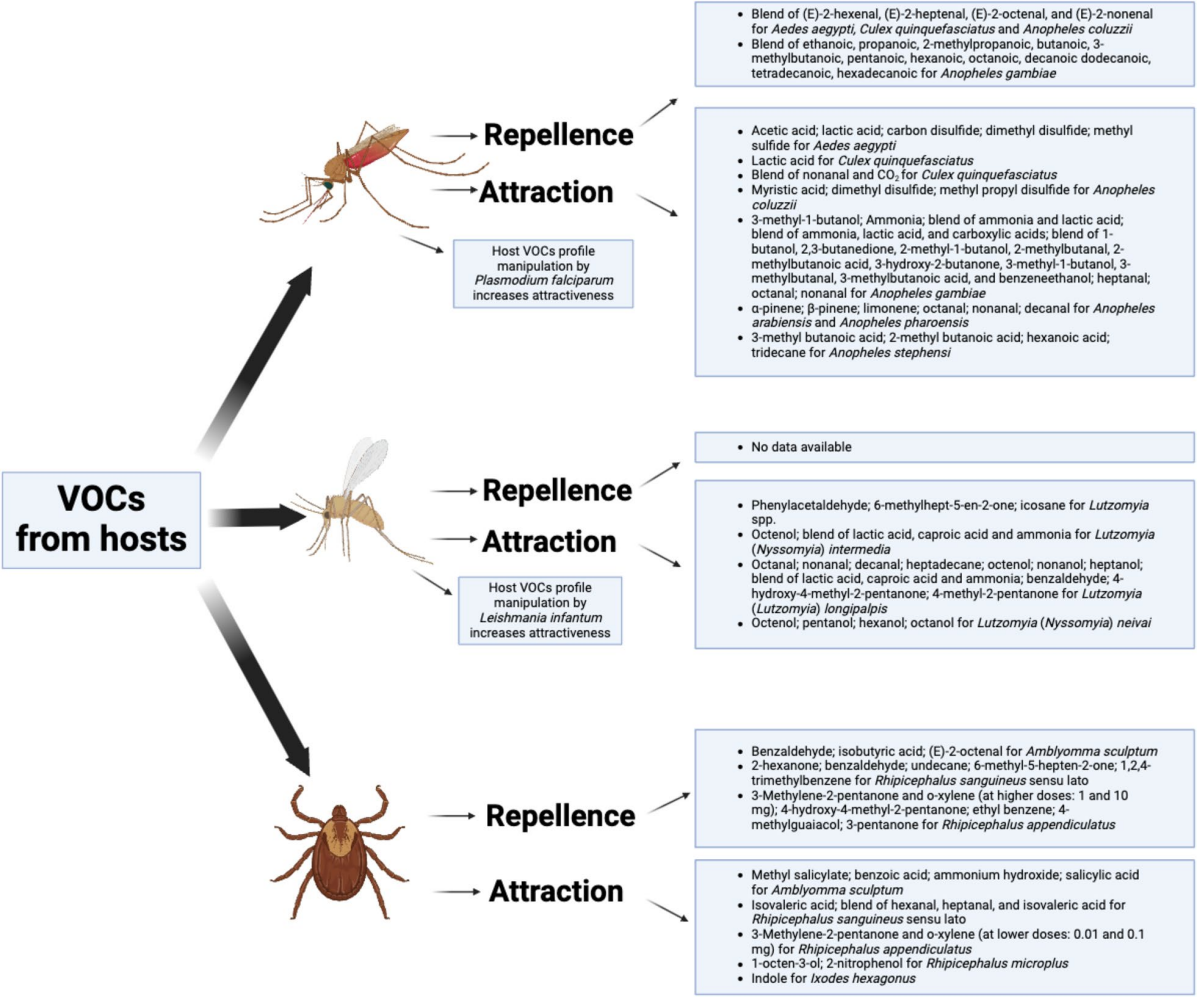
Host-borne volatile organic compounds (VOCs) influencing the olfactory behaviour of mosquitoes, sand flies, and ticks

**Fig. 2:**
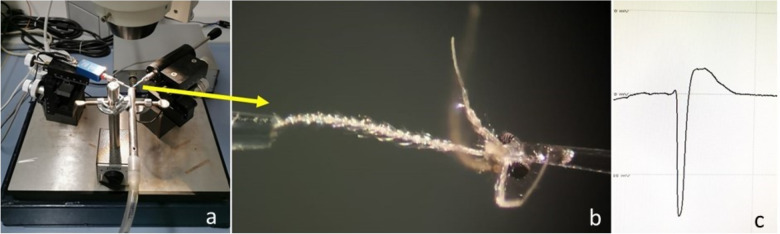
Electroantennography for the evaluation of sand flies chemoreceptivity to VOCs

**Fig. 3:**
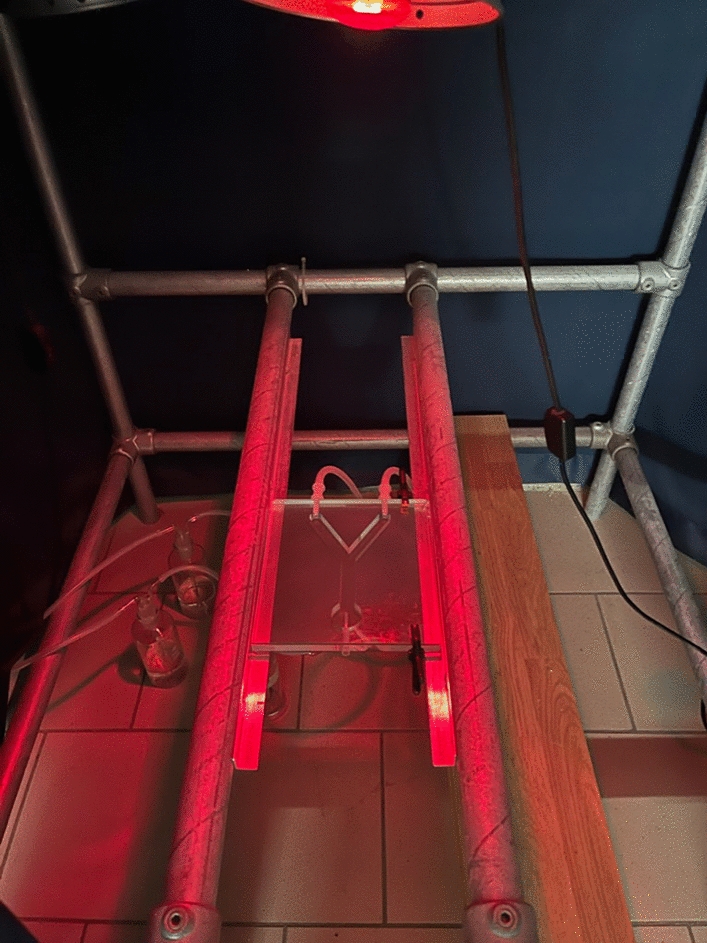
Y-tube olfactometer used for behavioural experiments with sand flies and other arthropods

In general, the host preference of blood feeding arthropod vectors is often investigated through the analysis of blood meal by molecular methods [31–38], or by host-choice experiments in laboratory conditions [39–42]. However, while the above methods are useful to investigate the host preference, they are limited in assessing the factors influencing the attracting capacity of the hosts. Conversely, the interaction between arthropod vectors and vertebrate hosts could be assessed through olfactory cues by determining the attractiveness or repellent effects of VOCs emitted by the hosts (Fig. 1). This is usually investigated at laboratory scale, collecting VOCs from the host through classical extraction methods; this can be done relying to adsorption materials used in solid-phase micro extraction (SPME) and direct-contact sorptive extraction (DCSE). Then, the collected compounds are subsequently identified through gas chromatography (GC), nuclear magnetic resonance (NMR) and mass spectrometry (MS) approaches [43]. Once identified, electroantennography (EAG/EAD; Fig. 2), also coupled with GC and MS (i.e., GC-EAD and GC–MS-EAD) can be used to evaluate which molecules of a VOCs’ bouquet are really perceived by a given arthropod vector. Electroantennography-active VOCs can be therefore tested in behavioural experiments such as olfactometer assays (e.g., Y-tube olfactometer and flight tunnel; Fig. 3) and in field/semi-field tests to shed light on their ecological role and potential use in vector monitoring and management.

Several studies on VOCs and their role as attractants or repellents have been performed for different arthropod vectors including mosquitoes [22, 42, 44–60], ticks [61–67], phlebotomine sand flies [68–76], triatomines [12], and tsetse flies [77, 78]. The latter includes species of the genus *Glossina*, which are exclusively found in Africa, where field and laboratory studies have been performed and reviewed elsewhere [78]. In the present review, we analysed current knowledge on the host-borne VOCs acting as attractants or repellents for worldwide distributed vectors of major medical and veterinary importance, such as mosquitoes, phlebotomine sand flies, and ticks. Moreover, we discuss the applied implications of these compounds, including their potential usefulness for an early diagnosis of vector-borne diseases (VBDs).

## Volatile organic compounds and mosquitoes

Mosquitoes are the most important arthropod vectors worldwide transmitting many deadly pathogens of human and veterinary concern, such as chikungunya, dengue, zika, West Nile virus, Rift Valley fever virus, Japanese encephalitis virus, *Plasmodium* spp., and *Dirofilaria* spp. [79–81]. Studies on the repellent and attraction effects of these arthropods using olfactory cues have been mostly assessed for *Anopheles* spp., *Aedes aegypti*, and *Culex* spp., which are vectors of deadly human pathogens [45, 47, 51, 82–87]. For example, the repellent effect of some VOCs isolated from cattle has been showed for *Ae. aegypti*, *Culex quinquefasciatus* and *Anopheles coluzzii* [22]. In the above study, unsaturated aldehydes (Table 1) extracted from the hair of Holstein heifer cattle showed a repellent effect against these insects under laboratory and field conditions [22]. In laboratory assays, the four-component blends (i.e., (*E*)-2-hexenal, (*E*)-2-heptenal, (*E*)-2-octenal, and (*E*)-2-nonenal) repelled females with an effect similar to that of commercial repellents that were used as control (i.e., *N, N*-diethyl-m-toluamide [DEET], ethyl butylacetylaminopropionate [IR3535], *p*-menthane-3, 8-diol [PMD], icaridin, and *d*-allethrin). Moreover, under field conditions, traps treated with the four-component blend captured significantly lesser mosquitoes in a site (average reduction of 84.8%) in comparison with the control traps (baited with CO_2_ and host kairomone), whereas in the second site the average reduction was of 55.5% [22].

**Table 1:** Attraction and repellent effects of volatile organic compounds (VOCs) associated with vertebrate hosts for mosquitoes, sand flies, and ticks

Arthropod group	Attractants	Reppellents	Refs.
Mosquitoes			
* Aedes aegypti*	Acetic acid; lactic acid; carbon disulfide; dimethyl disulfide; methyl sulfide	blend of (*E*)-2-hexenal, (*E*)-2-heptenal, (*E*)-2-octenal, and (*E*)-2-nonenal (laboratory and field experiments)	[22, 89]
* Culex quinquefasciatus*	Lactic acid; blend of nonanal and CO_2_	blend of (*E*)-2-hexenal, (*E*)-2-heptenal, (*E*)-2-octenal, and (*E*)-2-nonenal (Laboratory and field experiments)	[22, 86, 89]
* Anopheles coluzzii*	Myristic acid; dimethyl disulfide; methyl propyl disulfide	blend of (*E*)-2-hexenal, (*E*)-2-heptenal, (*E*)-2-octenal, and (*E*)-2-nonenal (laboratory and field experiments)	[22, 89]
* Anopheles gambiae*	3-methyl-1-butanol; ammonia; blend of ammonia and lactic acid; blend of ammonia, lactic acid, and carboxylic acids (ethanoic, propanoic, 2-methylpropanoic, butanoic, 3-methylbutanoic, pentanoic, hexanoic, octanoic, decanoic, dodecanoic, tetradecanoic, hexadecanoic); heptanal, octanal, nonanal; blend of ammonia, lactic acid, tetradecanoic acid; blend of 1-butanol, 2,3-butanedione, 2-methyl-1-butanol, 2-methylbutanal, 2-methylbutanoic acid, 3-hydroxy-2-butanone, 3-methyl-1-butanol, 3-methylbutanal, 3-methylbutanoic acid, and benzeneethanol	2-phenylethanol; blend of ethanoic, propanoic, 2-methylpropanoic, butanoic, 3-methylbutanoic, pentanoic, hexanoic, octanoic, decanoic dodecanoic, tetradecanoic, hexadecanoic	[45, 47, 48, 50, 51]
* Anopheles arabiensis; Anopheles pharoensis*	α-pinene; β-pinene; limonene; octanal; nonanal; decanal	–	[114]
*Anopheles stephensi*	3-methyl butanoic acid; 2-methyl butanoic acid; hexanoic acid; tridecane	–	[126]
Sand flies			
* Lutzomyia* (*Nyssomyia*) *intermedia*; *Lutzomyia migonei*; *Lutzomyia* (*Nyssomyia*) *whitmani*; *Lutzomyia fischeri*; *Lutzomyia shannoni*	Phenylacetaldehyde; 6-methylhept-5-en-2-one; icosane	–	[72]
* Lutzomyia* (*Nyssomyia*)* intermedia*	Octenol; blend of lactic acid, caproic acid and ammonia	–	[69]
* Lutzomyia* (*Lutzomyia*) *longipalpis*	Octanal; nonanal; decanal; heptadecane; octenol; nonanol; heptanol; blend of lactic acid, caproic acid and ammonia; benzaldehyde; 4-hydroxy-4-methyl-2-pentanone; 4-methyl-2-pentanone	–	[69, 73, 74, 103]
* Lutzomyia* (*Nyssomyia*)* neivai*	Octenol; pentanol; hexanol; octanol	–	[70, 71]
Ticks			
* Amblyomma sculptum*	Methyl salicylate; benzoic acid; ammonium hydroxide; salicylic acid	benzaldehyde; isobutyric acid; (*E*)-2-octenal	[108, 109]
* Rhipicephalus sanguineus* sensu lato	Isovaleric acid; blend of hexanal, heptanal, and isovaleric acid	2-hexanone; benzaldehyde; undecane; 6-methyl-5-hepten-2-one; 1,2,4-trimethylbenzene	[63, 66, 67]
* Rhipicephalus appendiculatus*	3-methylene-2-pentanone and o-xylene (at lower doses: 0.01 and 0.1 mg)	3-methylene-2-pentanone and o-xylene (at higher doses: 1 and 10 mg); 4-Hydoxy-4-methyl-2-pentanone; ethyl benzene; 4-methylguaiacol; 3-pentanone	[64]
* Rhipicephalus microplus*	1-octen-3-ol; 2-nitrophenol	–	[61]
* Ixodes hexagonus*	Indole	–	[62]

The olfactory preferences of mosquitoes may differ according to the species, being an important cue for understanding the host choice and behaviour of these arthropod vectors [88]. Indeed, the attractive effect of VOCs has been assessed for *Ae. aegypti*, *Cx. quinquefasciatus*, and *Cx. nigripalpus*, demonstrating that a higher number of compounds associated with bovine and avian blood (Table 1) were attractive for *Ae. aegypti* and, to a lesser extent for the other two species (Table 1) [89]. Again, lactic acid, a compound present in vertebrate hosts, was demonstrated to be attractive to *Ae. aegypti* [89, 90] and *Cx. quinquefasciatus* [89] but did not elicit any behavioural response for *Anopheles gambiae* mosquitoes [45, 91]. Nevertheless, the attractiveness of carbon dioxide to *An. gambiae* was significantly increased when combined with lactic acid in experimental assays [83], suggesting that although this compound is not attractive on its own, it may potentialize the response of other compounds present on the skin [83]. Indeed, the synergism among VOCs is an important factor to be considered when studying the role of these compounds in the behaviour of arthropod vectors, as demonstrated for *An. gambiae* mosquitoes, for which ammonia, lactic acid, and a mixture of carboxylic acids (Table 1) were tested alone and in combination to assess the olfactory responses of this mosquito species [45, 47, 51]. While ammonia was found to be attractive, the mixture of carboxylic acids was repellent, whereas lactic acid did not attract the mosquitoes. However, when ammonia, carboxylic acids and lactic acid were combined, they were significantly more attractive for *An. gambiae* mosquitoes, than ammonia alone [45, 47, 51]. In addition, the component blends from the above-mentioned studies became even more attractive to *An. gambiae* by the addition of butan-1-amine (associated with CO_2_), and 3-methyl-1-butanol [92]. Similarly, nonanal and CO_2_ synergized in field experiments in which traps containing the two compounds captured significantly more *Cx. quinquefasciatus* mosquitoes than traps baited only with CO_2_ [86], therefore suggesting that host volatiles may have different effects on the host seeking behaviour of arthropods, according to their abundance and combination.

Though not emitted by hosts, some VOCs have also been associated with the selection of oviposition sites by *An. gambiae*, *An. arabiensis* and *Ae. aegypti* gravid females [19, 93, 94], demonstrating that compounds produced in water bodies containing mosquito larvae may influence the selection of oviposition sites [93]. For example, dimethyl disulfide and dimethyl trisulfide detected in emanations from water containing fourth instar larvae (L4) acted as oviposition repellents, also causing retention of eggs within the gravid females [93]. On the other hand, VOCs (i.e., nonane and 2,4-pentanedione) identified in emanations containing first instar larvae (L1) were considered attractants, being the presence of these two compounds in water related with significantly higher oviposition rates when compared to untreated water in semi-field trials [93]. The findings above indicate that the selection of the oviposition site by mosquitoes may be mediated by chemical compounds, and that the presence of larvae instars may impact on the choice of oviposition site by female mosquitoes, with L1 causing an attractive effect, and L4 repellent and inhibition outcomes on oviposition [93]. The oviposition selection sites of *Ae. aegypti* are also affected by VOCs produced in water bodies containing immature stages [19]. Indeed, by using blends of bioactive VOCs identified in eggs (i.e., 2,4-dimethylhept-1-ene; 2,6-dimethyl-7-octen-2-ol; camphor; decanal), in second instar larvae (L2) (i.e., 4-cyanocyclohexene; (*E*)-2-octenal; nonanal; decanal 4-(2-methylbutan-2-yl) phenol), in L4 (i.e., 2,4-dimethylhept-1-ene; (*E*)-2-heptanal; nonanal; camphor; (*E*)-2-nonenal; (*E*)-2-decenal; 4-(2-methylbutan-2-yl) phenol), and in pupae exuviae (4-cyanocyclohexene; 2,6-dimethyl-7-octen-2-ol; nonanal) of *Ae. aegypti* it was demonstrated that gravid females were more stimulated to lay eggs in response to VOCs produced in water bodies containing late-stage larvae [19]. All the above suggested that VOCs may signalize the density of conspecific aquatic stages, influencing on the oviposition site choice by gravid females, which may avoid laying eggs on sites in which the odour indicates high density of conspecific competitors [19].

## Volatile organic compounds and sand flies

Sand flies are involved in the transmission of viruses (e.g., Phleboviruses: Naples, Sicilian and Toscana viruses; [95]) bacteria (e.g., *Bartonella bacilliformis*; [96]), and most importantly, of protozoa of the genus *Leishmania* to humans and animals [97]. The latter is transmitted by sand flies of the genus *Phlebotomus* spp. and *Lutzomyia* spp. in the old and new world, respectively [98]. Studies on the interaction among sand fly species and their hosts have been performed worldwide, and they mainly focused on the evaluation of blood meal by molecular analysis in these insects [36, 99–101]. Besides the detection of blood meal, the interaction of sand flies with vertebrate hosts may also be assessed by determining the attractiveness or repellent effect of these insects by VOCs, though few investigations are available. *Lutzomyia* species (members of subgenera *Lutzomyia* (*Lu.*) and *Nyssomyia* (*N.*)) were tested with VOCs from humans, dogs, and foxes [68, 69, 72–74]. Of the 42 VOCs identified from the hair of 33 human male volunteers in Brazil, seven compounds (i.e., Phenylacetaldehyde; 6-methylhept-5-en-2-one; tetradecane; pentadecane; hexadecane; nonadecane; icosane) were tested for the attractiveness of field captured *Lutzomyia* spp. (i.e., *n* = 420 specimens; 75.4% *Lutzomyia* (*N.*) *intermedia*; 4.5% *Lutzomyia migonei*; 2.8% *Lutzomyia* (*N.*) *whitmani*; 0.9% *Lutzomyia fischeri*; 0.2% *Lutzomyia shannoni*; and 16.2% damaged sand flies non-identified) [72]. In the study above, four VOCs were demonstrated to induce a significant activation response in sand flies (i.e., phenylacetaldehyde; 6-methylhept-5-en-2-one; pentadecane; icosane), while three of them (i.e., Phenylacetaldehyde; 6-methylhept-5-en-2-one; icosane) were attractive to female sand flies [72]. *Lutzomyia* (*Lu.*) *longipalpis* also had a behavioural response to VOCs found on humans, with the compounds octenol, nonanol and heptanol activating and attracting male and female sand flies in behavioural experiments [73]. Additionally, *Lu.* (*Lu.*) *longipalpis* and *Lu.* (*N.*) *intermedia* were attracted to octenol and a synthetic human odour BG-Mesh Lure^™^ (BGML—lactic acid, caproic acid and ammonia) in field experiments using CDC light traps baited with these compounds [69]. The attractiveness of VOCs from the skin of different human individuals for *Lu.* (*Lu.*) *longipalpis* has also showed that there is a significant variation in individual attractiveness of human subjects to sandflies. However, the attractants were not identified in that study [68].

Volatile organic compounds emitted by *L. infantum* infected dogs were also assessed to test the responses of *Lu.* (*Lu.*) *longipalpis* [74], demonstrating that, when tested individually, octanal, nonanal, decanal and heptadecane activated and attracted male sand flies, whereas only decanal and nonanal showed an activation response of females [74]. Moreover, the blend of octanal, decanal and heptadecane increased both the activation and attraction behaviour in males, while the mixture of octanal and decanal acted only in the activation [74]. The higher attractiveness of these VOCs to male *Lu.* (*Lu.*) *longipalpis* may be related to the biology of this sand fly species, since males are also known to be attracted by the host odours, and under field studies they have been demonstrated to arrive first at the host sites than females [9]. Furthermore, females of this species have been suggested to be more attracted to the host volatile compounds when in combination with male-produced sex pheromone, a strategy for easing blood meal [8, 10]. In contrast to VOCs released from infected dogs, *Lu.* (*Lu.*) *longipalpis* females were demonstrated to be significantly more attracted than males in response to skin emanations from humans under experimental conditions. However, the attractive factors were not identified, advocating further experiments to confirm this observation [102]. In wildlife, a single study on VOCs from foxes (*Vulpes vulpes*) demonstrated that female *Lu.* (*Lu.*) *longipalpis* were attracted by the natural odour, and by a synthetic blend mimicking the odour of this host, as well as by individual compounds (i.e., benzaldehyde; 4-hydroxy-4-methyl-2-pentanone; 4-methyl-2-pentanone) identified in the fox odour [103].

The attraction of VOCs has also been tested for the species *Lu.* (*N.*) *neivai*, a vector of *Leishmania* (*Viannia*) *brasiliensis* in South America [70, 71], in which octenol [70, 71], pentanol, hexanol, and octanol [71] acted both in activating and attracting field captured female sand flies in behavioural experiments using wind tunnel. The above findings suggest that research on the attractiveness of sand flies by VOCs from mammalian hosts are relevant to assess the host preference of these insects, as well as to enhance the efficiency of conventional traps used for monitoring and controlling phlebotomine sand flies.

The effect of VOCs in sand fly selection of oviposition sites has also been studied for *Phlebotomus papatasi*, in which volatile semiochemicals produced by bacterial isolates (i.e., Actinobacteria, Bacteroides, Firmicutes, and Proteobacteria) had an attractive response for gravid females at low doses (i.e., 10^7^ cells per ml), and a repellent response at higher concentrations (10^9^ cells per ml) [104]. Though, the identification of the attractive VOCs produced by these bacteria was not completed, data above suggest that the attractiveness of *Ph. papatasi* to those VOCs is dose dependent.

## Volatile organic compounds and ticks

Ticks are important vectors of pathogens of zoonotic concern worldwide, being able to transmit infectious agents to humans, domestic animals, and wildlife [80, 105]. These arthropods are responsible for significant economic losses to the livestock industry, which are directly related with the decrease in animal production and, indirectly, with the transmission of pathogens such as *Anaplasma* spp., *Babesia* spp., and *Theileria* spp. [105, 106]. For this reason, studying the host preference, the pathogen transmission times [107] as well as the mechanisms behind the resistance of some breeds to ticks, is paramount to improve control measures against these ectoparasites.

Studies on VOCs have been employed in the assessment of the attractive and repellent effects [108] of these compounds for ticks (Table 1). For example, the attraction of some volatiles (e.g., methyl salicylate; benzoic acid; ammonium hydroxide; salicylic acid) has been tested in nymphs of *Amblyomma sculptum* ticks collected on naturally infected horses from Brazil [108]. These data suggested that such compounds may be used as trapping methods for *A. sculptum* ticks [108]. The same tick species has also been demonstrated to be repelled by benzaldehyde and isobutyric acid, which, in turn, are compounds previously detected in tick resistant mammalian hosts [63, 108]. Moreover, in another study performed on donkeys, the (*E*)-2-octenal was demonstrated to act as a repellent for this tick species [109]. Of notice, the above compound was found in sebum extracts of donkeys, but not of horses, being the latter less resistant to the infestation by *A. sculptum* ticks. Such data suggested that the compound (*E*)-2-octenal could be used as a repellent for reducing *A. sculptum* populations in susceptible hosts [109].

In dogs, VOCs have also been assessed in breeds resistant to *Rhipicephalus sanguineus* sensu lato ticks to detect compounds with potential repellent effect [63, 66]. For example, beagles have been demonstrated to be less susceptible to tick infestation when compared to English cocker spaniel breed [110], resulting in a lower attractiveness of beagles’ odour to *R. sanguineus* s.l. [111]. The observation above was further investigated through the isolation of VOCs from the skin of these two breeds, in which the gas chromatography analysis detected a greater number of compounds on odour extracts collected from beagles than in the extracts from cocker spaniel [63]. In addition, among the compounds detected in beagles, three of the most abundant (i.e., 2-hexanone; benzaldehyde; undecane), showed repellent effect at the behavioural assays [63]. These data confirmed that beagles produce a larger amount of potentially natural repellents against this tick species [63]. This was further supported by another study, in which the VOCs produced by the above-mentioned breeds were compared with the ones produced by the miniature pinscher, which is a putative tick-resistant breed [66]. In the aforementioned study, 2-hexanone and benzaldehyde were detected in the odour extracts of all three breeds, with 6-methyl-5-hepten-2-one (sulcatone), and 1,2,4-trimethylbenzene being more abundant in miniature pinscher dogs. In addition, the overall abundance of these compounds was significantly higher in beagles and miniature pinschers than in cocker spaniels [66]. In the behavioural assays, the compounds 6-methyl-5-hepten-2-one and 1,2,4-trimethylbenzene were repellent for ticks, supporting that both may play a role in the resistance of these breeds to *R. sanguineus* s.l. ticks [66].

The attractiveness of VOCs from dogs has also been assessed in a study identifying compounds that may be involved in host attraction and localization by *R. sanguineus* s.l. [67]. In the above study isovaleric acid, hexanal, heptanal, and sulcatone significantly stimulated the olfactory receptors of female ticks using the single sensillum recording technique [67], while in the Y-tube olfactometer bioassays the ticks were attracted only to isovaleric acid and to a blend of hexanal, heptanal, and isovaleric acid [67]. Overall, those data suggested that the latter compounds may be involved in the host location by *R. sanguineus* s.l. [67].

## Volatile organic compounds and vector-borne pathogens

The association of VOCs with pathogens transmitted by arthropods have been assessed for *Plasmodium falciparum* [112–115] and for *L. infantum* [75, 76, 116, 117], transmitted by mosquitoes and sand flies, respectively (Fig. 1). Some VOCs attractive for mosquitoes have been suggested to be produced by *P. falciparum* as a strategy of this parasite for facilitating its transmission [113, 118–123]. For example, the compound (*E*)-4-hydroxy-3-methyl-but-2-enyl pyrophosphate (HMBPP) produced by *P. falciparum* has been suggested to increase the production and release of *An. gambiae* attractants from blood of malaria infected individuals, increasing the likelihood of vector bite in infected humans [121]. Indeed, VOCs already known to attract mosquitoes (i.e., α-pinene and 3-carene) were detected at significantly higher levels in the breath of malaria infected children as compared with uninfected ones [123]. Nevertheless, it was later demonstrated that HMBPP does not alter the levels of the mosquito attractant α-pinene, indicating that the mechanism used by *Plasmodium* spp. parasites to increase attractiveness of infected individuals remains unclear [124]. Moreover, under field conditions, a blend of VOCs (i.e., α-pinene; β-pinene; limonene; octanal; nonanal; decanal) reflecting the odours induced by *P. falciparum* gametocyte parasitizing the red blood cells, attracted 2.5 times more *Anopheles* spp. mosquitoes than non-baited traps, indicating that the parasite could manipulate the host-seeking behaviour of malaria vectors [114]. Similarly, experimental trials demonstrated that the aldehydes heptanal, octanal, and nonanal, which are produced in larger amount by infected individuals, enhanced an increased attractiveness of *An. gambiae* vectors [125], consequently affecting the transmission of the parasite.

The attraction of *Anopheles stephensi* mosquitoes to mice infected by *Plasmodium chabaudi* has also been demonstrated in an experimental study, which also showed a clear difference in the VOCs composition between infected and non-infected individuals, identifying 11 VOCs (i.e., N,N-dibutylformamide; tridecane; 2-pyrrolidone; 3-methyl-2-buten-1-ol; 3-methyl butanoic acid; 2-hexanone; benzaldehyde; and 4 unidentified compounds) as indicators of infection during the chronic phase [126]. In addition, 3-methyl butanoic acid, 2-methyl butanoic acid, hexanoic acid, and tridecane were attractive for the mosquitoes, whereas benzothiazole, which decreased in infected mice, caused a significant reduction in mosquito attraction [126].

Volatile organic compounds of symptomatic and asymptomatic malaria human patients has been used as biomarkers for the diagnosis of this disease, demonstrating that some compounds (e.g., 4-hydroxy-4-methylpentan-2-one; nonanal; toluene; 2-ethylhexan-1-ol; ethylbenzene; ethylcyclohexane; propylcyclohexane; hexane) were predictors of malaria infection [127]. Importantly, these volatiles presented a sensitivity of 100% in patients without clinical signs, suggesting their potential in screening of malaria infected individuals [127].

Similarly, VOCs production profile of *L. infantum* infected vs non-infected dogs has been assessed for the identification of possible biomarkers for the diagnosis of leishmaniasis [116]. In the above study, some VOCs (i.e., octanal; nonanal; undecane; β-hydroxyethylphenyl ether; decanal; tetradecane; nonyl cyclopentane; 8-pentadecanone; heptadecane; 2-ethylhexyl-salicylate) were identified being selectively expressed with different profiles in infected (with and without clinical signs) vs non-infected dogs [116]. In particular, β-hydroxyethylphenyl ether, nonanal, heptadecane, 2-ethylhexyl-salicylate, decanal, and octanal showed a significant variation in production, which could be regarded as potential biomarkers of infection by *L. infantum* in dogs [116].

The differences in VOCs profiles from *L. infantum* infected and non-infected individuals also influenced the attractiveness of sand flies, under experimental studies both in dogs [75], and hamsters for *Lu.* (*Lu.*) *longipalpis* [117]. The above studies demonstrated that male and female of *Lu.* (*Lu.*) *longipalpis* were equally attracted to the odour of uninfected dogs as compared to a solvent control [75]. Conversely, blood seeking females were significantly more attracted than males when exposed to the odour of *L. infantum* infected dogs [75]. Similarly, female *Lu.* (*Lu.*) *longipalpis* were significantly more attracted to Golden Hamsters (*Mesocricetus auratus*) experimentally infected by *L. infantum*, than to uninfected individuals [117]. Finally, the attractiveness of infected vs non-infected dogs to sand flies was also assessed for *Ph. perniciosus* and *Ph. perfiliewi* under experimental and field conditions, demonstrating that male and female insects were significantly more attracted to dogs infected by *L. infantum* than to non-infected [76]. Nevertheless, the VOCs profile of the animals in the above study was not assessed. Overall, this scientific evidence supports the concept that the parasite may modify the profile of VOCs on the host to make it more attractive to sand fly vectors.

## Conclusions 

The olfactory cues of arthropod vectors have been mainly studied for mosquitoes and ticks, with the identification of attractive and repellent VOCs emanated by their vertebrate hosts, and in a lesser extent for phlebotomine sand flies in which up to date, only attractive VOCs have been assessed (Table 1). The potential of these compounds to influence the behaviour of these arthropods have been assessed in several studies, being proved as a key factor in the host choice by ticks, mosquitoes, and sand flies. These investigations are of great importance to better understand the host preference of some vectors by assessing the VOCs production profile, which differ not only according with the different host species, but also within hosts of the same species, affecting the individual susceptibility to arthropod bites [128, 129]. In addition, such studies are also useful for improving strategies of monitoring and control, particularly under the current global warming scenario, which also affects arthropod chemical ecology [130].

The differences in the production of VOCs in infected vs non-infected hosts, indicate that vector-borne pathogens such as *P. falciparum* and *L. infantum* may modify the profile of these compounds in infected hosts to make them more attractive for blood seeking vectors, which consequently increases the odds of transmission of VBDs. Such intriguing ecological hypothesis remains less explored for ticks and tick-borne pathogens. Under the above circumstances, the identification of VOCs biomarkers produced by infected host cells could be a potential tool for the diagnosis of vector borne pathogens. Nevertheless, the complexity of the skin microenvironment of vertebrate hosts should be considered when evaluating the VOCs production. For example, infections by skin dwelling microorganisms (e.g., bacteria, dermatophytes, mites), may influence the composition of VOCs on the host, potentially increasing the attraction to arthropod vectors [131, 132]. The above may be also true for non-infectious diseases such as cancer, which are also known to alter the VOCs profile of the hosts [28]. However, whether the conditions above increase the risk of infection by vector borne pathogens should be further investigated.

Volatile organic compounds may also be useful for improving the mass rearing of arthropod vectors by using compounds that mimic the odour of the hosts, which could be applied in artificial membranes to increase the acceptability of arthropods to artificial feeding systems; therefore, avoiding the use of live animals in the mass rearing of blood feeding arthropods. Finally, another future perspective for the use of VOCs is the potential integration between compounds emitted by hosts and vector pheromones, which may be used as a strategy for the monitoring of arthropod vectors through the increase in the attractiveness of trapping methods. All the applications above could be potentially useful towards reducing the impact of VBDs of medical and veterinary relevance.

## Data Availability

Not applicable.

## Abbreviations

VOCs: Volatile organic compounds
VBDs: Vector-borne diseases
CO_2_: Carbon dioxide
SPME: Solid-phase micro extraction
DCSE: Direct-contact sorptive extraction
GC: Gas chromatography
NMR: Nuclear magnetic resonance
MS: Mass spectrometry
EAG: Electroantennography
EAD: Electroantennographic detection
HMBPP: (*E*)-4-hydroxy-3-methyl-but-2-enyl pyrophosphate
